# Supplementary material for: The CHARGE syndrome-associated protein FAM172A controls AGO2 nuclear import
Source: Life Sci Alliance. 2023 May 23;6(8):e202302133. doi: 10.26508/lsa.202302133 (PMC10205598; doi:10.26508/lsa.202302133)
Supplement: Supplementary file 9 [file LSA-2023-02133_TableS1.docx]

**Table S1. List of primers used in this study**

|  | **Oligos Name** | **Sens** | **Sequence 5’-3’** |
| --- | --- | --- | --- |
| Genotyping | *FAM172A^Tp^* | F  R | 5’-GGGAGTAAGTCCTACCAATGTTAAATC  5’-TGACCTTCTAAACAGTCCCATATCCCC |
|  | *Wild type* | F  R | 5’-GAAGTGGGAACAAAACACCCTTGG  5’-GTAAGGCTCGCACTGACATAGC |
| mutagenesis | Gibson for FAM172A in backbone pCDNA3.1 | N-term-F  C-term-R | 5’-CTCGGCATGGACGAGCTGTACAAGGAATTCATGAAAAAGGACGAACCACCTT  TT  5’-CACTATAGAATAGGGCCCTCTAGACTCACAGCTCCTCGTGC |
|  | Gibson for FAM172A in backbone pIRES2-GFP | N-term-F  C-term-R | 5’-TTTCTGAAGAAGATCTGAGATCTCTCGAGATGAAAAAGGACGAACCACCTTTT  5’-GGATCCCGCGGGTCGACCTGGAGGTCACAGCTCCTCGTGC |
|  | FAM172A [mNLS] | N-term-R  mNLS-F  mNLS-R  C-term-F | 5’-CCTTTCCTGTTGCCCTGCTGGCTCATCTG  5’-CAACAGGAAAGGAGAGATAAGGTCTCCAAGGAAACAAAGAAGCAACAGGAT  TTCATGAGAAG  5’-ATCCTGTTGCTTCTTTGTTTCCTTGGAGACCTTATCTCTCCTTTCCTGTTGC  CCTGTGGCTC  5’-AAGCAACAGGATTTCTATGAGAAGTACCGCAACCC |
|  | FAM172A[P-] | N-term-R  P [-] -F  P [-] -R  C-term-F | 5’-ATGCATTTTCTGCTTTTCCACTTCTATATAGTTTTCATTTGGGTT  5’-GAAGTGGAAAAGCAGAAAATGCATAAACAGTCATCATCTTCTCAAGGTACACA  GGAGCCAGCAGGGAAG  5’-TTCCCGCTTCCCTGCTGGCTCCTGTGTACCTTGAGAAGATGATGACTGTTTAT  GCATTTTCTGCTTTTCCACTTC  5’-GATGAGCCAGCAGGGAAGCGGGAAAGGAGAGATAAG |
|  | FAM172A[P+] | N-term-R  P [+] -F  P [+] -R  C-term-F | 5’-ATGCATTTTCTGCTTTTCCACTTCTATATAGTTTTCATTTGGGTT  5’-GAAGTGGAAAAGCAGAAAATGCATAAACAGGACGATGATGACGATGGTGACG  ATGAGCCAGCAGGGAAG  5’-TTCCCGCTTCCCTGCTGGCTCATCGTCACCATCGTCATCATCGTCCTGTTTAT  GCATTTTCTGCTTTTCCACTTC  5’-GATGAGCCAGCAGGGAAGCGGGAAAGGAGAGATAAG |
|  | _FLAG_AGO1[WT] | 3xFLAG-F  3xFLAG-R  Ago1-F  Ago1-R | 5’-TACGACTCACTATAGGGAGACCCAATGGACTACAAAGACCATGACG  5’-GGGTCCCGCTTCCTTGTCATCGTCATCCTTGTAATC  5’-GACGATGACAAGGAAGCGGGACCCTCG  5’-GGTGACACTATAGAATAGGGCCCTTCAAGCGAAGTACATGGTGC |
|  | _FLAG_AGO2[WT] | 3xFLAG-F  3xFLAG-R  Ago2-F  Ago2-R | 5’-TACGACTCACTATAGGGAGACCCAATGGACTACAAAGACCATGACG  5’-CTGGAATGGGTGCTTGTCATCGTCATCCTTGTAATC  5’-GACGATGACAAGCACCCATTCCAGTGGTGTAAC  5’-CTTTTTTGGATCAGCAAAGTACATGGTGCGC |
|  | _FLAG_AGO2[+NLS] | +NLS-F  +NLS-R | 5’-ATGTACTTTGCTGGTGGTGATCCAAAAAAGAAGAGAAAGGTAGATCCAAAAA  AGAAGAGAAAGGTAGATCCAAAAAAGAAGAGAAAGGTATGAAGGGCCCTATTCTATAGTGTCACC  5’-GGTGACACTATAGAATAGGGCCCTTCATACCTTTCTCTTCTTTTTTGGATCTAC  CTTTCTCTTCTTTTTTGGATCTACCTTTCTCTTCTTTTTTGGATCACCACCAGCAAAGTACAT |
| qPCR | *Cd44*_vE8-9 | F  R | 5’-TACCCCAGTTTTTCTGGATCAGG  5’-GCCATCCTGGTGGTTGTCTG |
|  | *Cd44*_vE13-14 | F  R | 5’-TGGAAGACTTGAACAGGACAGG  5’-GTTTTCGTCTTCTTCCGGCTC |
|  | *Cd44*_cE4-5 | F  R | 5’-ACAGACCTACCCAATTCCTTCG  5’-GGGTGCTCTTCTCGATGGTG |
