# Supplementary material for: The CHARGE syndrome-associated protein FAM172A controls AGO2 nuclear import
Source: Life Sci Alliance. 2023 May 23;6(8):e202302133. doi: 10.26508/lsa.202302133 (PMC10205598; doi:10.26508/lsa.202302133)
Supplement: Supplementary file 10 [file LSA-2023-02133_TableS2.docx]

**Table S2: List of antibodies used in this study**

|  | **Antibody Name** | **Dilution** | **Source** | **RRID** |
| --- | --- | --- | --- | --- |
| Primary antibodies | Rat monoclonal Anti-AGO1 | WB (1:1000)  IF (1:500)  IP (3 μg/ml) | Sigma SAB4200084 | AB_10602786 |
|  | Rabbit polyclonal Anti-AGO2 | WB (1:1000)  IF (1:200)  IP (3 μg/ml) | Abcam ab32381 | AB_867543 |
|  | Mouse monoclonal Anti-AGO2 | WB (1:1000)  IF (1:200)  IP (2μg/ml) | Abcam ab57113 | AB_2230916 |
|  | Rabbit polyclonal Anti-FAM172A | WB (1:1000)  IF (1:500)  IP (2 μg/ml) | Abcam ab1213 | 64 AB_11127114 |
|  | Mouse monoclonal Anti-FLAG tag | WB (1:1000)  IP (2μg/ml) | Sigma F1804 | AB_262044 |
|  | Mouse monoclonal Anti-GAPDH | WB (1:3000) | Santa Cruz Biotech sc-32233 | AB_627679 |
|  | Rabbit polyclonal Anti-GFP | WB (1:3000)  IF (1:500) | Abcam ab290 | AB_303395 |
|  | Mouse monoclonal Anti-HA tag | WB (1:1000) | Biolegend 901502 | AB_2565007 |
|  | Rabbit polyclonal Anti-Ki67 | IF (1:1000) | Abcam ab15580 | AB_443209 |
|  | Rabbit polyclonal Anti-KPNA2 | WB (1:5000) | Bethyl A300-484A | AB_451019 |
|  | Rabbit polyclonal Anti-KPNB1 | WB (1:2000) | Bethyl A301-803A | AB_1233062 |
|  | Rabbit polyclonal Anti-KPNB3 | WB (1:200) | Santa Cruz Biotech sc-55527 | AB_2127684 |
| Secondary antibodies | Rabbit polyclonal Anti-Lamin A/C | WB (1:1000) | Abcam ab227176 | - |
|  | Rat monoclonal Anti-MBP tag | WB (1:1000)  IP (2μg/ml) | Covance Research Products MRT-131P | AB_10720557 |
|  | Mouse monoclonal Anti-MYC tag | WB (1:500)  IF (1:100) | In house hybridoma  (9E10) | - |
|  | Goat polyclonal Anti-MYC tag | WB (1:1000) | Abcam ab9132 | AB_307033 |
|  | Rabbit polyclonal Anti-Nucleolin | WB (1:1000)  IP (2μg/ml) | Abcam ab50279 | AB_881762 |
|  | Donkey Alexa Fluor 488 Anti-Rat IgG | IF (1:500) | Jackson  ImmunoResearch 712-545-150 | AB_2340683 |
|  | Donkey Alexa Fluor 594 Anti-Rabbit IgG | IF (1:500) | Jackson  ImmunoResearch  711-585-152 | AB_2340621 |
|  | Donkey Alexa Fluor 647 Anti-Mouse IgG | IF (1:500) | Jackson  ImmunoResearch  715-605-150 | AB_2340862 |
|  | Goat Anti-Rat IgG HRP | WB (1:10000) | Santa Cruz Sc-2032 | AB_631755 |
|  | Horse Anti-Mouse IgG HRP | WB (1:2000) | Cell signaling 7076 | AB_330924 |
|  | Goat Anti-Rabbit IgG HRP | WB (1:500) | Abcam 6721 | AB_955447 |
|  | Rabbit Anti-Goat IgG HRP | WB (1:25000) | Bio-Rad 1721034 | AB_11125144 |
